# Simvastatin-induced cell cycle arrest through inhibition of STAT3/SKP2 axis and activation of AMPK to promote p27 and p21 accumulation in hepatocellular carcinoma cells

**DOI:** 10.1038/cddis.2016.472

**Published:** 2017-02-23

**Authors:** Sin-Ting Wang, Hsiu J Ho, Jaw-Town Lin, Jeng-Jer Shieh, Chun-Ying Wu

**Affiliations:** 1 Institute of Biomedical Sciences, National Chung Hsing University, Taichung, Taiwan; 2 Division of Gastroenterology, Taichung Veterans General Hospital, Taichung, Taiwan; 3 School of Medicine, Fu Jen Catholic University, Taipei, Taiwan; 4 Institute of Population Health Sciences, National Health Research Institutes, Miaoli, Taiwan; 5 Department of Education and Research, Taichung Veterans General Hospital, Taichung, Taiwan; 6 Department of Life Sciences and Rong Hsing Research Center for Translational Medicine, National Chung Hsing University, Taichung, Taiwan; 7 Faculty of Medicine, School of Medicine, National Yang-Ming University, Taipei, Taiwan; 8 Department of Public Health and Graduate Institute of Clinical Medical Science, China Medical University, Taichung, Taiwan; 9 National Institute of Cancer Research, National Health Research Institutes, Miaoli, Taiwan

## Abstract

Hepatocellular carcinoma (HCC) is characterized by a poor prognosis and is one of the leading causes of cancer-related death worldwide. Simvastatin, an HMG-CoA reductase inhibitor, which decreases cholesterol synthesis by inhibiting mevalonate pathways and is widely used to treat cardiovascular diseases. Simvastatin exhibits anticancer effects against several malignancies. However, the molecular mechanisms underlying the anticancer effects of simvastatin on HCC are still not well understood. In this study, we demonstrated simvastatin-induced G0/G1 arrest by inducing p21 and p27 accumulation in HepG2 and Hep3B cells. Simvastatin also promoted AMP-activated protein kinase (AMPK) activation, which induced p21 upregulation by increasing its transcription. Consistent with this finding, we found genetic silencing of AMPK reduced p21 expression; however, AMPK silencing had no effect on p27 expression in HCC cells. Simvastatin decreased Skp2 expression at the transcriptional level, which resulted in p27 accumulation by preventing proteasomal degradation, an effect mediated by signal transducer and activator of transcription 3 (STAT3) inhibition. Constitutive STAT3 activation maintained high-level Skp2 expression and lower level p27 expression and significantly prevented G0/G1 arrest in simvastatin-treated HCC cells. Mevalonate decreased simvastatin-induced AMPK activation and rescued phospho-STAT3 and Skp2 expression in HCC cells, which resulted in the prevention of G0/G1 arrest through inhibition of p21 and p27 accumulation. Moreover, simvastatin significantly decreased tumor growth in HepG2 xenograft mice. Consistently, we found that simvastatin also increased p21 and p27 expression in tumor sections by reducing Skp2 expression and inducing AMPK activation and STAT3 suppression in the same tumor tissues. Taken together, these findings are demonstrative of the existence of a novel pathway in which simvastatin induces G0/G1 arrest by upregulating p21 and p27 by activating AMPK and inhibiting the STAT3–Skp2 axis, respectively. The results identify novel targets that explain the beneficial anticancer effects of simvastatin treatment on HCC *in vitro* and *in vivo*.

Hepatocellular carcinoma (HCC) is the fifth most common cancer and the third leading cause of cancer-related mortality worldwide.^1, 2^ HCC is induced by multiple conditions, including hepatitis B virus (HBV) infection, hepatitis C virus (HCV) infection, alcoholic liver disease and metabolic syndrome.^3^ Despite advances in HCC diagnosis and treatment, most HCC patients still have a poor prognosis because of tumor progression or tumor recurrence.^4^ Therefore, it is essential to develop chemopreventive strategies to improve HCC patient outcomes. In a previous study of patients with HBV- and HCV-related HCC, we reported that antiviral therapy reduced HCC recurrence and mortality after liver resection or radiofrequency ablation.^5, 6, 7^ However, antiviral therapy only reduces the risk of HCC. It does not eliminate the risk of the disease. Metabolic syndrome seems to have important roles in HCC development in the post-antiviral therapy era. We found that the risk of HCC was significantly higher in diabetic patients than in the general population and that metformin decreased the risk of HCC by inhibiting hepatoma cell proliferation and inducing cell cycle arrest.^8^ Statins have been suggested to inhibit HCC progression and increase HCC survival in patients with hyperlipidemia.

Statins are 3-hydroxy-3-methylglutaryl coenzyme A (HMG-CoA) reductase inhibitors, which catalyze the rate-limiting step in cholesterol biosynthesis and are widely used to treat patients with hypercholesterolemia.^9^ The chemopreventive effects of statins have been reported in several cancers, such as gastric, breast, colon, liver and prostate cancer.^10, 11, 12^ Their potential mechanisms of action involve the inhibition of tumor cell proliferation, the promotion of cell cycle arrest, the induction of apoptotic cell death, and the inhibition of cell migration, invasion and metastasis.^9, 10, 11, 12, 13^ In addition, statins attenuate the production of isoprenoid units, which are critical for the activation of Rho, Ras and Rab proteins.^12, 13^ Statins also affect cyclin-dependent kinase inhibitors^12^ and have been shown to inhibit PI3K/AKT signaling and induce derepression of PTEN expression to inhibit breast cancer cell growth.^14, 15^ AMP-activated protein kinase (AMPK), a cellular energy sensor that mediates metabolic homeostasis under environmental stress conditions, was recently reported to be activated by statins.^16, 17, 18, 19, 20^ Autophagy inhibition enhances the anticancer effects of statins in digestive malignancies.^18, 21^ AMPK activation has been reported to suppress cell proliferation in non-tumor and tumor cells by regulating cell cycle progression or inhibiting protein synthesis.^9, 22, 23, 24^ In addition, signal transducer and activator of transcription 3 (STAT3), an important signaling protein that contributes to HCC development and progression, may be inhibited by statins in several cellular systems.^25, 26, 27^ The findings of recent studies suggest that simvastatin may inhibit cancer cell growth by inducing apoptosis or cell cycle arrest at the G0/G1 phase, thereby decreasing the risk of HCC.^9, 28^ However, whether AMPK and STAT3 have roles in the anticancer effects of statins in HCC remains unclear.

In this study, we showed that simvastatin can induce cell cycle arrest and increase cyclin-dependent kinase inhibitor expression in HCC cells. We demonstrated that simvastatin-induced cell cycle arrest was regulated by AMPK activation and STAT3 inactivation to transcriptionally increase p21 expression and stabilize p27 protein expression by inhibiting Skp2 expression, respectively. Moreover, we established a HepG2 tumor-bearing xenograft animal model to examine the antitumor effects of simvastatin *in vivo*. Our results showed that simvastatin inhibited tumor growth and that the expression patterns of p21, p27, Skp2, AMPK and STAT3 were similar to those of the *in vitro* study. Overall, our findings provide evidence of the existence of a novel molecular mechanism by which simvastatin exerts its anticancer effects in HCC.

## Results

### Simvastatin induces p21 and p27 expression-dependent G0/G1 cell cycle arrest in HCC cell lines

**Figure 1:**
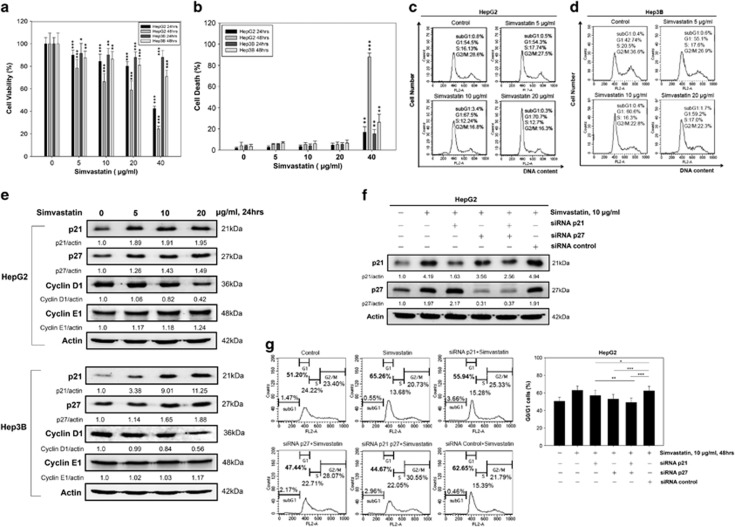
Simvastatin induces p21 and p27-dependent G0/G1 cell cycle arrest in HCC cell lines. Simvastatin suppressed cell growth in HCC cells. HepG2 and Hep3B cells were treated with various concentrations of simvastatin for 24 and 48 h. (**a**) Cell growth inhibition was measured by CCK-8 assay. (**b**) Cell death rates were determined by viable cell counting. These data are presented as percentages of vehicle-treated cells (*un-treated *versus* treated of HepG2 or Hep3B cells for 24 h or 48 h). (**c** and **d**) Simvastatin-induced HCC cell G0/G1 phase arrest. HepG2 and Hep3B cells were treated with simvastatin (0, 5, 10 or 20 *μ*g/ml) for 48 h, and then cell cycle distributions were analyzed by PI staining and flow cytometry. (**e**) Simvastatin-induced G0/G1 phase-related protein expression in HCC cells. The cells were treated with simvastatin (0, 5, 10 or 20 *μ*g/ml) for 24 h, and then the cell lysates were harvested for analysis of the expression of the cell cycle-related proteins p21, p27, cyclin D1, cyclin E1 and *β*-actin by immunoblotting. (**f** and **g**) Simvastatin-induced p21- and p27-dependent G0/G1 cell cycle arrest in HCC cells. HepG2 cells were transfected with p21, p27 or control siRNA for 24 h and then treated with 10 *μ*g/ml simvastatin for 48 h. The cells were then harvested, and their DNA content and protein expression were analyzed by flow cytometry and immunoblotting using p21, p27 and *β*-actin antibodies. Data are expressed as the mean±S.E.M. of three independent experiments. Statistically significant differences between the un-treated and treated groups are indicated. *P<0.05, **P<0.01, ***P<0.001

To determine whether simvastatin influences cell growth in hepatoma, we investigated the effect of simvastatin on cell viability in the HepG2 and Hep3B hepatoma cell lines. Simvastatin had significant dose- and time-dependent inhibitory effects on hepatoma cell growth in HepG2 and Hep3B cells, as demonstrated by CCK-8 assay (Figure 1a). To evaluate whether simvastatin induces cell death in hepatoma, we performed a viable cell count assay by Trypan blue staining in HepG2 and Hep3B cells. The results showed that the decrease in HepG2 and Hep3B cell viability elicited by 5–20 *μ*g/ml simvastatin treatment (Figure 1a) did not correspond to the cell death rate of both cells subjected to the same dosage of simvastatin treatment (Figure 1b). Thus, simvastatin mainly inhibited HepG2 and Hep3B cell growth but did not cause cell death at doses <40 *μ*g/ml. In addition, simvastatin treatment at a dose of 40 *μ*g/ml induced higher apoptosis in HepG2 cells than in Hep3B cells (Supplementary Figure S1). The mechanism underlying statin-induced p53-dependent apoptosis has been clearly elucidated.^29^ Here, we focused on how simvastatin inhibits hepatoma cell growth. To investigate the mechanism underlying simvastatin-induced hepatoma cell growth inhibition, we analyzed the effect of simvastatin on cell cycle distribution using flow cytometry. We observed that after 48 h of incubation, simvastatin arrested cell cycle progression at the G0/G1 phase in HepG2 cells in a dose-dependent manner. The sizes of the sub-G1 populations of HepG2 cells were not significantly increased in the control and simvastatin-treated groups (Figure 1c). Simvastatin had a similar effect on cell cycle distribution in Hep3B cells (Figure 1d). As shown in Figure 1c, various doses of simvastatin arrested the cell cycle at the G0/G1 phase. The cell populations increased from 54.5% in control cells to 70.7% (20 *μ*g/ml simvastatin) in HepG2 cells and from 42.74% in control cells to 59.2% (20 *μ*g/ml simvastatin) in Hep3B cells. In addition, we observed that simvastatin treatment decreased cyclin D1 expression and increased p21 and p27 expression but had no significant modulatory effects on cyclin E1 expression in either HepG2 or Hep3B cells (Figure 1e). To determine whether simvastatin treatment-induced G0/G1 cell cycle arrest was dependent on p21 or p27 expression in HCC cells, we genetically silenced p21 and p27 in HepG2 cells and confirmed the decline of p21 and p27 expression by immunoblotting (Figure 1f). These p21 and p27 knockdown cells rescued the G0/G1 cell population under simvastatin treatment (Figure 1g). These results indicated that simvastatin treatment-induced G0/G1 cell cycle arrest that was dependent on p21 and p27 accumulation in HepG2 cells.

### Simvastatin induces p21 and p27 accumulation by increasing p21 transcription and preventing p27 degradation in HepG2 cells

**Figure 2:**
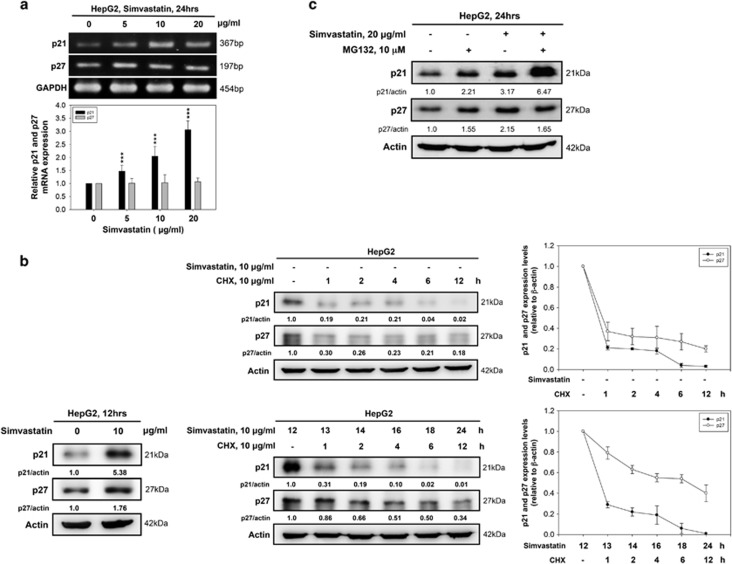
Simvastatin-induced p21 and p27 protein upregulation was associated with transcriptional activation and protein degradation inhibition, respectively. (**a**) Simvastatin modulated p21 mRNA expression. HepG2 cells were treated with simvastatin (0, 5, 10 or 20 *μ*g/ml) for 24 h, and p21, p27 and GAPDH mRNA expression levels were detected by RT-PCR and real-time PCR. (**b**) The effects of p21 and p27 protein stability in simvastatin-treated HCC cells. HepG2 cells were treated with 10 *μ*g/ml CHX alone for 1, 2, 4, 6 or 12 h or 10 *μ*g/ml simvastatin for 12 h. After 12 h, simvastatin-treated cells were co-treated with 10 *μ*g/ml CHX for 1, 2, 4, 6 or 12 h. The cell lysates were harvested to detect p21, p27 and *β*-actin protein expression by immunoblotting. The intensity of each protein signal was determined by ImageJ software (downloaded from the NIH website (http://rsb.info.nih.gov/ij)). (**c**) Inhibition of proteasomal degradation promoted p21 accumulation, but not p27 accumulation, in simvastatin-treated HCC cells. HepG2 cells were treated with 20 *μ*g/ml simvastatin with or without 10 *μ*M MG132 for 24 h and then subjected to immunoblotting for the detection of p21, p27 and *β*-actin expression levels. Data are expressed as the mean±S.E.M. of three independent experiments. Statistically significant differences between the un-treated and treated groups are indicated. **P<0.01, ***P<0.001

To evaluate whether simvastatin increased p21 and p27 levels at the transcriptional, translational or degradation level, we first analyzed p21 and p27 mRNA levels by RT-PCR and real-time PCR after simvastatin treatment. We observed that p21 mRNA levels were upregulated and that p27 mRNA levels were not significantly affected by simvastatin stimulation (Figure 2a). We then examined whether simvastatin stabilized p21 and p27 protein expression. Compared with non-treated cells, simvastatin-treated cells exhibited a slower decrease in p27 expression than in p21 expression following treatment with the translation inhibitor cycloheximide (CHX) (Figure 2b). One of the mechanisms that controls p21 and p27 levels is proteasomal degradation.^30, 31^ We used MG132 to clarify whether simvastatin inhibits p21 and p27 protein degradation by blocking the ubiquitin proteasome pathway. We surmised that if simvastatin inhibits p21 and p27 proteasomal degradation, the protein levels of p21 and p27 will not change in the presence of MG132, irrespective of simvastatin administration. We thus also surmised that if simvastatin does not inhibit proteasomal degradation, the protein levels of p21 and p27 will increase upon simvastatin treatment in the presence of MG132, which would indicate that simvastatin either inhibits protein degradation by blocking another pathway, such as the lysosomal pathway, or induces protein synthesis at the transcriptional and/or translational level. Based on the results of this experiment, we determined that p21 levels increased significantly in the presence of MG132 following simvastatin treatment, but p27 levels were not affected by simvastatin treatment (Figure 2c). Taken together, our results suggested that simvastatin-induced p21 protein expression mainly at the transcription level and upregulated p27 protein expression predominantly by preventing protein degradation in HepG2 cells.

### Simvastatin-induced p21 transcriptional upregulation is AMPK dependent in HepG2 cells

**Figure 3:**
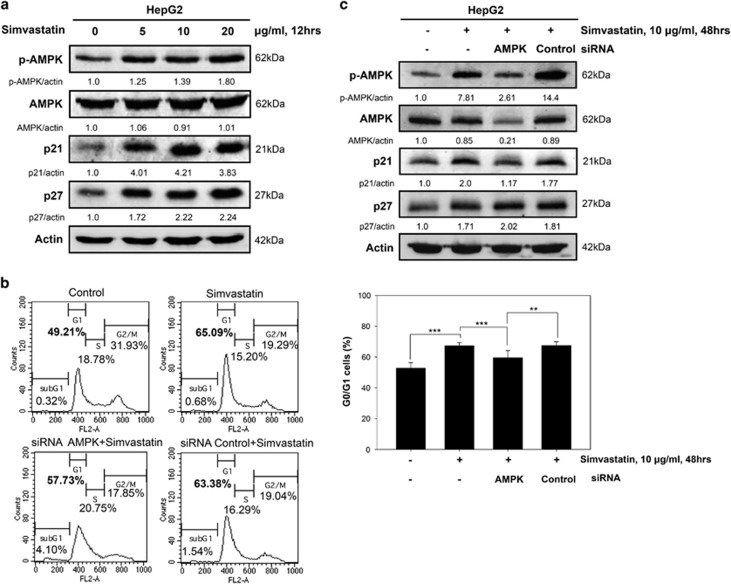
Simvastatin-induced AMPK activation and p21 upregulation partially induced G0/G1 arrest in HepG2 cells. (**a**) Simvastatin-induced AMPK activation was associated with p21 and p27 upregulation. HepG2 cells were treated with simvastatin (0, 5, 10 or 20 *μ*g/ml) for 12 h, and then immunoblotting was used to detect p-AMPK, AMPK, p21, p27 and *β*-actin protein expression levels. (**b** and **c**) Genetic silencing of AMPK reduced G0/G1 phase arrest and p21 expression in simvastatin-treated HCC cells. HepG2 cells were transfected with AMPK or control siRNA for 24 h and then treated with 10 *μ*g/ml simvastatin for 48 h. (**b**) Cells were collected for analysis of their DNA content by flow cytometry. (**c**) Cell lysates were harvested to detect protein expression levels by immunoblotting using p-AMPK, AMPK, p21, p27 and *β*-actin antibodies. The results were obtained from three independent experiments. Data are expressed as the mean±S.E.M. of three independent experiments. **P<0.01, ***P<0.001

Simvastatin has been reported to activate the AMPK pathway.^18, 19^ To investigate whether AMPK has a role in influencing G0/G1 phase arrest in HepG2 cells after simvastatin treatment, we detected AMPK expression levels by immunoblotting analysis. Similar to the above results pertaining to the expression patterns of p21 and p27, these results showed that simvastatin treatment increased the level of phosphorylated AMPK (Figure 3a). To further examine whether AMPK-mediated simvastatin-induced G0/G1 phase arrest, we genetically knocked down AMPK expression in HepG2 cells using small interfering RNA (siRNA). AMPK knockdown cells displayed a partially but significantly decreased G0/G1 cell population under simvastatin treatment (Figure 3b). We also found that AMPK knockdown reduced p21 expression, but not p27 expression, in simvastatin-treated HepG2 cells (Figure 3c). These results provided evidence that AMPK activation by simvastatin-induced p21 expression at the transcriptional level and that p21 expression may be involved in simvastatin-induced cell cycle G0/G1 arrest in hepatoma cells.

### Simvastatin transcriptionally inhibits Skp2 expression and promotes p27 accumulation in HepG2 cells

**Figure 4:**
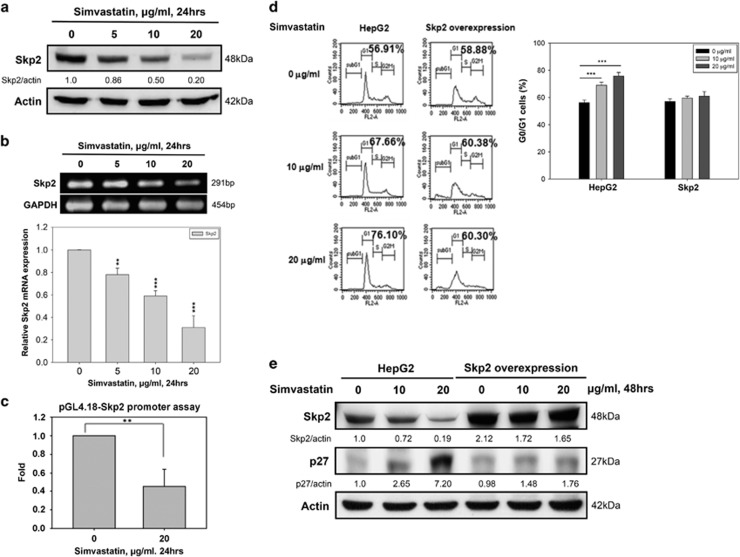
Simvastatin-induced p27 upregulation was Skp2-dependent and promoted G0/G1 phase cell cycle arrest in HepG2 cells. (**a**) Simvastatin decreased Skp2 protein expression in HepG2 cells. HepG2 cells were treated with simvastatin (0, 5, 10 or 20 *μ*g/ml) for 24 h, and then cell lysates were harvested to detect the protein expression levels of Skp2 and *β*-actin by immunoblotting. (**b**) Simvastatin inhibited Skp2 mRNA expression in HepG2 cells. After the same treatment, cells were collected for analysis of Skp2 and GAPDH mRNA expression levels by RT-PCR and real-time PCR. (**c**) Simvastatin reduced Skp2 promoter activity. HepG2 cells were transfected with a pGL4.18-Skp2 promoter plasmid for 24 h and were then treated with simvastatin (0 or 20 *μ*g/ml) for 24 h. The cell lysates were harvested to assay luciferase activity using a dual-luciferase assay kit. Data were normalized to Renilla luciferase activity and expressed as fold inductions of the control. (**d**) Skp2 overexpression rescued cells from simvastatin-induced G0/G1 cell cycle arrest. Control and Skp2-overexpressing HepG2 cells were treated with simvastatin (0, 10 or 20 *μ*g/ml) for 48 h, and then the cells were collected for DNA content assay by flow cytometry. (**e**) Simvastatin was unable to change Skp2 and p27 protein expression levels in Skp2-overexpressing HepG2 cells. Control and Skp2-overexpressing HepG2 cells were treated with simvastatin (0 or 20 *μ*g/ml) for 24 h, and then the cell lysates were collected for protein expression detection by immunoblotting using Skp2, p27 and *β*-actin antibodies. The results were obtained from three independent experiments. Data are expressed as the mean±S.E.M. of three independent experiments. **P<0.01, ***P<0.001

Recent studies have shown that Skp2 E3 ligase activity can promote p27 degradation to prevent p27-induced cell cycle arrest.^30, 32, 33^ Therefore, we hypothesized that simvastatin may inhibit Skp2 to promote p27 accumulation and G0/G1 cell cycle arrest in HCC cells. As shown in Figure 4a, simvastatin decreased Skp2 protein expression in a dose-dependent manner. To evaluate the mechanism underlying simvastatin-induced decreases in Skp2 expression in HepG2 cells, we first analyzed Skp2 mRNA expression after simvastatin treatment using RT-PCR and real-time PCR. Our data showed that Skp2 mRNA expression decreased during simvastatin treatment (Figure 4b). We also confirmed that simvastatin inhibited Skp2 transcription in HepG2 cells by Skp2 promoter-driven luciferase assay (Figure 4c). Consistent with these findings, we found that Skp2 overexpression significantly prevented simvastatin-induced G0/G1 cell cycle arrest and p27 accumulation (Figures 4d and e). In addition, compared with control HepG2 cells, Skp2-overexpressing HepG2 cells did not maintain the simvastatin-enhanced p27 protein stability after treatment with CHX (Supplementary Figure S2). These results indicated that simvastatin treatment downregulated Skp2 expression at the transcriptional level and then promoted p27 accumulation to induce G0/G1 cell cycle arrest.

### Simvastatin inhibits the STAT3/Skp2 axis to induce G0/G1 cell cycle arrest in HepG2 cells

**Figure 5:**
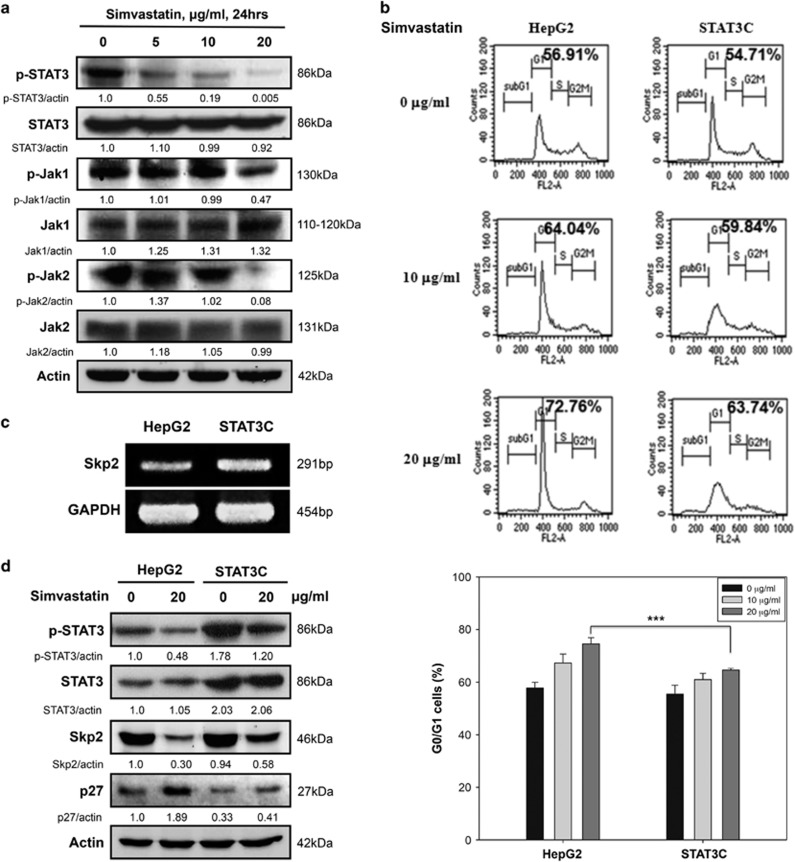
STAT3C mutants maintained Skp2 expression to prevent p27 accumulation and G0/G1 cell cycle arrest in simvastatin-treated HepG2 cells. (**a**) Simvastatin inhibited the Jak1/Jak2-STAT3 pathway in HCC cells. HepG2 cells were treated with simvastatin (0, 5, 10 or 20 *μ*g/ml) for 24 h, and then the cell lysates were harvested for the detection of protein levels by immunoblotting using p-Jak1, Jak1, p-Jak2, Jak2, p-STAT3, STAT3 and *β*-actin antibodies. (**b**) STAT3C mutants prevented simvastatin-induced G0/G1 cell cycle arrest in HepG2 cells. HepG2 cells were stably transfected with control vectors or STAT3C mutants and were then treated with simvastatin (0, 10 or 20 *μ*g/ml) for 48 h. Then, the cells were collected for DNA content assay by flow cytometry. (**c**) Constitutive STAT3 activation in HepG2 cells upregulated Skp2 expression at the transcriptional level. Control and constitutive STAT3 activity levels in HepG2 cells were analyzed by RT-PCR to determine HepG2 Skp2 mRNA expression levels. (**d**) HepG2 cells stably expressing STAT3C maintained higher Skp2 levels and lower p27 levels. Control and STAT3C-expressing HepG2 cells were treated with simvastatin (0 or 20 *μ*g/ml). Twenty-four hours later, the cell lysates were collected to detect protein expression by immunoblotting using p-STAT3, STAT3, Skp2, p27 and *β*-actin antibodies. The results were obtained from three independent experiments. Data are expressed as the mean±S.E.M. of three independent experiments. ***P<0.001

It has been reported that STAT3 inactivation induces Skp2 downregulation and p27 upregulation in cervical and gastric cancer.^34, 35^ We investigated the molecular mechanisms underlying this phenomenon to determine whether STAT3 interacts with the Skp2/p27 pathway in simvastatin-treated HepG2 cells. We found that simvastatin decreased phospho-STAT3 levels, as well as those of its upstream regulators, Jak1 and Jak2, in HepG2 cells, as shown in Figure 5a. Next, we attempted to evaluate whether constitutive STAT3 activation facilitated by the expression of a constitutively activate mutant of STAT3 (STAT3C) could reverse the above simvastatin-induced effects. We transfected HepG2 cells with the STAT3C expression vector and selected cells that stably expressed STAT3C. We observed that the G0/G1 cell population in STAT3C-transfected cells was lower than that in mock cells after simvastatin treatment (Figure 5b). As shown in Figure 5c, the mRNA expression levels of Skp2 increased significantly in STAT3C-transfected cells compared with those in mock cells, confirming the existence of a relationship between STAT3 and Skp2. To further investigate whether cell cycle-related molecule expression levels were affected in the mock and STAT3C-transfected groups after simvastatin treatment, we detected p-STAT3, STAT3, Skp2 and p27 expression levels by immunoblotting analysis. We found that STAT3C-transfected cells treated with simvastatin maintained higher Skp2 protein expression levels but displayed severely decreased p27 expression compared with mock cells (Figure 5d). Thus, our results indicated that simvastatin-induced p27 upregulation is Skp2 dependent and occurs through inhibition of the STAT3/Skp2 activation axis.

### Mevalonate reverses the activation of AMPK and the inhibition of STAT3 facilitated by simvastatin treatment in HepG2 cells

**Figure 6:**
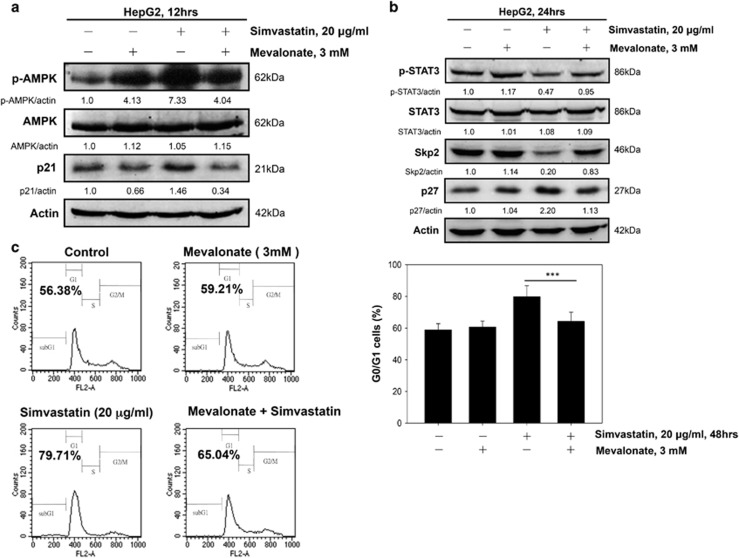
Exogenous mevalonate suppressed the inhibitory effects of simvastatin on cell growth by reversing the simvastatin-induced AMPK activation and STAT3 inhibition in HepG2 cells. (**a**) Mevalonate inhibited simvastatin-induced AMPK activation and p21 enhancement. HepG2 cells were pre-treated with or without mevalonate (3 mM) for 1 h and then treated with 20 *μ*g/ml simvastatin for 12 h. The cells were collected to detect p-AMPK, AMPK, p21 and *β*-actin protein levels by immunoblotting. (**b**) Mevalonate blocked simvastatin-induced STAT3 inhibition and Skp2 depletion. HepG2 cells were pre-treated with or without mevalonate (3 mM) for 1 h and then treated with 20 *μ*g/ml simvastatin for 24 h. The cell lysates were harvested to analyze protein levels by immunoblotting using p-STAT3, STAT3, Skp2, p27 and *β*-actin antibodies. (**c**) Mevalonate inhibited simvastatin-induced G0/G1 cell cycle arrest. HepG2 cells were pre-treated with or without mevalonate (3 mM) for 1 h, followed by treatment with 20 *μ*g/ml simvastatin for 48 h. The cells were collected for analysis of DNA content by flow cytometry using PI staining. Results are shown as the mean±S.E.M. of three independent experiments. ***P<0.001

Many studies have demonstrated that statins decrease cholesterol biosynthesis by inhibiting the endogenous mevalonate pathway.^36, 37, 38^ Here, restoration of mevalonate, the HMG-CoA reductase product, significantly reduced AMPK phosphorylation (Figure 6a) and maintained higher phospho-STAT3 and Skp2 levels, with decreased p21 and p27 accumulation, in simvastatin-treated HepG2 cells (Figure 6b). Moreover, we also observed that mevalonate treatment effectively prevented simvastatin-induced G0/G1 cell cycle arrest in HepG2 cells (Figure 6c). Taken together, our findings demonstrated that AMPK pathway activation and STAT3/Skp2 pathway inhibition in HepG2 cells are dependent on inhibition of the conversion of HMG-CoA reductase to mevalonate by simvastatin.

### Simvastatin inhibits tumor growth in xenograft animal models

**Figure 7:**
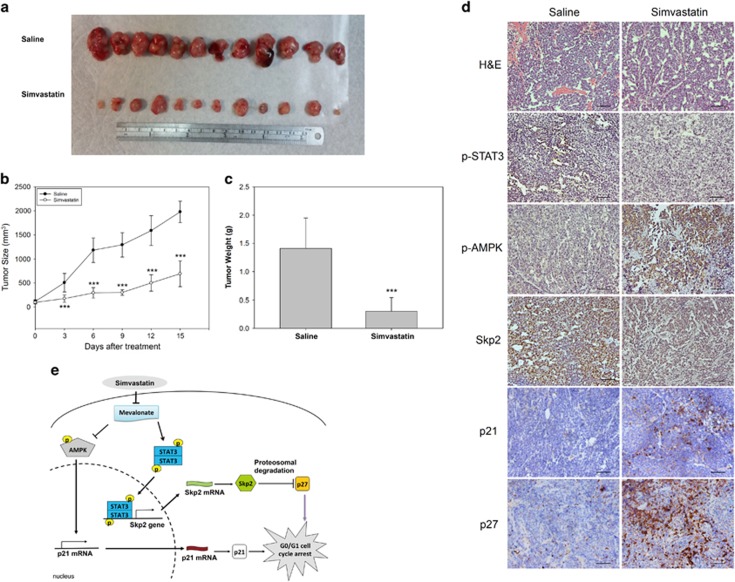
Simvastatin inhibited HepG2 tumor growth in xenograft mice. HepG2 tumor-bearing BALB/c nude mice were divided into control and simvastatin treatment groups. The mice were treated with saline or 20 mg/kg body weight simvastatin twice a day by intraperitoneal (i.p.) injection. (**a**) Tumor tissues from the saline and simvastatin treatment groups were harvested at 14 days after injection. These tissues were isolated from each mouse after killing. (**b**) Tumor growth curves derived from nude mice in the saline- and simvastatin-treated groups. The tumor volumes of the nude mice were calculated twice a day for 2 weeks. (**c**) The tumor weights of the nude mice were measured after killing. Results are shown as the mean±S.E.M (*n*=12). Saline-treated mice compared with simvastatin-treated mice. ***P<0.001 (**d**) IHC analysis using antibodies against human p21, p27, Skp2, p-AMPK and p-STAT3 in the tumor tissues of the saline- and simvastatin-treated groups. All scale bars are 50 *μ*m. (**e**) To summarize the *in vitro* and *in vivo* results of this study, we found that simvastatin promoted G0/G1 cell cycle arrest by increasing p21 and p27 expression via AMPK pathway activation and STAT3/Skp2 pathway suppression, respectively. These phenomena were dependent on inhibiting the production of mevalonate by simvastatin treatment in the HCC model

To examine the antitumor effect of simvastatin *in vivo*, we injected HepG2 cells subcutaneously into the flanks of BALB/c nude mice to establish a HepG2 tumor-bearing animal model. As shown in Figure 7a, we demonstrated that simvastatin significantly suppressed tumor growth (Figure 7b) and reduced tumor weight (Figure 7c) in nude mice. Consistent with these findings, we found that p21 and p27 expression levels were increased by simvastatin treatment in mice compared with saline treatment (Figure 7d). In addition, using immunohistochemistry (IHC), we also observed that simvastatin treatment increased AMPK phosphorylation, reduced STAT3 phosphorylation and decreased Skp2 expression in tumor tissues (Figure 7d). Taken together, these results indicated that simvastatin repressed tumor growth by increasing p21 and p27 expression through AMPK activation and STAT3/Skp2 axis inhibition *in vivo*.

## Discussion

In this study, we investigated the molecular mechanisms by which simvastatin induces cell growth arrest in HCC cells and found that simvastatin treatment results in suppression of the oncoproteins STAT3 and Skp2 and activation of the energy sensor protein AMPK. We demonstrated that simvastatin-induced G0/G1 cell cycle arrest was regulated by AMPK activation and STAT3 inactivation to transcriptionally increase p21 expression and stabilize p27 protein expression by inhibiting Skp2 expression. In addition, we also showed that this effect could be recovered by treatment with mevalonate, the product of HMG-CoA reductase. In contrast, in a xenograft animal model, we found that simvastatin treatment significantly suppressed HepG2 tumor growth and tumor weight. The results of our tumor section evaluations by IHC showed that simvastatin treatment increased p21 and p27 expression and AMPK activation and decreased Skp2 expression and STAT3 phosphorylation. These data provide evidence of a novel mechanism explaining the beneficial anticancer effects of simvastatin. These results are summarized in Figure 7e.

AMPK has been shown to promote accumulation of p53, which upregulates the protein expression of p21 at the transcriptional level and phosphorylates p27 at T198 to increase protein stability, thereby causing G0/G1 phase arrest.^39, 40, 41, 42^ In our experiments, we observed that simvastatin activated AMPK and induced p21 and p27 expression to cause G0/G1 phase arrest in HepG2 (p53 wild-type) and Hep3B (p53 mutant) cells (Figures 1a and d). We also found that simvastatin-induced p21 expression at the transcriptional level and promoted p27 accumulation by preventing proteasomal degradation (Figure 2). However, genetic knockdown of AMPK did not reduce p27 protein expression levels in our study (Figure 3c). These results suggested that simvastatin may regulate p21 gene expression induced by AMPK activation via other transcriptional machinery that functions independently of p53 activity such as FoxO1- and/or FoxO3-mediated *p21* mRNA transcription in neonatal cardiomyocytes.^43^ Moreover, the above results suggest that simvastatin may not promote p27 protein accumulation through AMPK-mediated p27 phosphorylation at T198.

The p27 stability could be regulated by Skp2 and certain ubiquitin proteins.^30^ Previous studies have suggested that targeting Skp2 results in p27-mediated G0/G1 phase arrest.^43^ These findings indicate that Skp2 was crucial for regulating p27 expression in our models. Consistent with this finding, we observed that simvastatin decreased Skp2 expression at both the mRNA and the protein level concurrently with increases in p27 accumulation, resulting in G0/G1 phase arrest (Figures 4a and b). Skp2 overexpression reduced p27 protein levels and inhibited G0/G1 phase arrest in simvastatin-treated HepG2 cells (Figure 4d). The functions of Skp2 have been investigated, and previous studies have demonstrated that its expression can be regulated at the transcriptional level, as can its cell cycle-dependent degradation.^32, 44, 45^ In our study, we found that simvastatin decreased Skp2 expression mainly at the transcriptional level (Figure 4b) and that this effect may be mediated by STAT3 inhibition in HepG2 cells. Constitutive STAT3 activation maintained much higher levels of Skp2 and lower levels of p27 and reduced the G0/G1 phase cell population in simvastatin-treated HepG2 cells (Figures 5b and d). These results suggest that simvastatin inhibited the STAT3/Skp2 axis to promote p27 accumulation, resulting in G0/G1 phase arrest. Previous studies have shown that several mediators, including ERK1/2, E2F1, Akt, PPAR*γ*, FOXP3 and STAT3, regulate Skp2 expression at the transcriptional level.^35, 46, 47, 48, 49, 50^ Recent studies have also reported that simvastatin can inhibit ERK1/2, Akt and STAT3 expression to suppress tumor cell growth and induce cell apoptosis.^51, 52^ However, no reports regarding STAT3 and Skp2 regulation in simvastatin-induced growth arrest in HCC are available. In this study, we demonstrated that simvastatin suppressed HCC cell growth by reducing Skp2 expression to cause p27 accumulation and induced G0/G1 phase arrest via STAT3 inhibition. It is possible that simvastatin may act through other pathways, such as ERK1/2 and Akt, to decrease Skp2 expression. These ideas will be evaluated in the future.

Mevalonate is synthesized by HMG-CoA reductase, and mevalonate pathway dysregulation promotes oncogenic transformation.^53^ Statins are well-known HMG-CoA reductase inhibitors and have been reported to inhibit renal cancer cell growth and metastasis.^52^ A recent report also suggested that statins inhibit IL-6-induced STAT3 phosphorylation and that this effect can be reversed by mevalonate in human hepatocytes.^54^ However, no studies demonstrating that maintaining mevalonate levels can facilitate the maintenance of much higher STAT3/Skp2 activity levels and lower p27 levels to prevent G0/G1 phase arrest in statins-treated cells are available. In our study, we found that mevalonate treatment could recover STAT3 and Skp2 expression, reduce AMPK phosphorylation, and downregulate p27- and p21-mediated G0/G1 arrest in simvastatin-treated HepG2 cells (Figures 6a and b). Studies focusing specifically on AMPK have shown that some mevalonate downstream products are essential for cell proliferation and survival. Moreover, these studies have also indicated that the anticancer effects of statins on AMPK activation may be mediated, at least in part, through inhibition of this pathway.^23^ The mechanisms by which mevalonate abrogated statin-induced AMPK activation are important and need to be evaluated in future studies.

In this study, we demonstrated that simvastatin-induced p27- and p21-mediated growth arrest by inhibiting STAT3/Skp2 and activating AMPK in HCC cells. Consistent with this finding, we also observed that simvastatin treatment inhibited tumor growth and reduced tumor volume in a mouse xenograft model of human HepG2 tumors. Tumor tissues evaluated using IHC assay also showed that simvastatin treatment increased p21 and p27 expression, increased AMPK activation, decreased Skp2 expression and reduced STAT3 phosphorylation. Furthermore, previous clinical studies have reported that statin use is associated with relative reductions in the risks of colorectal cancer, breast cancer and prostate cancer.^55, 56^ These findings suggest that statins warrant further investigation in chemoprevention and therapeutic clinical trials. In our study population, patients were newly diagnosed with HCC and had undergone liver resection as their initial HCC therapy. In addition, these patients must have used statins for >80 days (Supplementary Figure S4). Data regarding the cumulative incidence of overall mortality are shown in Supplementary Figure S3. These data suggest that statin use was associated with significantly lower overall mortality in HCC patients after liver resection than statin non-use. Therefore, statin use as a clinical therapy in HCC patients may be warranted. The findings of this study serve as evidence of the possible usefulness of statins in HCC patients and suggest that statin use has clinical significance, as statins can reduce the incidence of tumor recurrence, enhance chemoprevention and increase overall survival.

## Materials and methods

### Reagents and antibodies

Simvastatin was purchased from Cayman (Ann Arbor, MI, USA) and propidium iodide (PI), CHX, MG132 and mevalonate were obtained from Sigma (St. Louis, MO, USA). TRIzol reagent was obtained from Invitrogen (Carlsbad, CA, USA). Antibodies specific to p21, p27, cyclin D1, cyclin E1, phospho-AMPK, AMPK, phospho-STAT3 and STAT3 were purchased from Cell Signaling Technology (Danvers, MA, USA), the antibody specific to p45^SKP2^ was purchased from Invitrogen, and the antibody specific to *β*-actin was purchased from Santa Cruz (Santa Cruz, CA, USA).

### Cell culture

Human hepatoma cell lines, HepG2 and Hep3B were cultured in Dulbecco's modified Eagles' medium (DMEM) supplemented with 10% fetal bovine serum (FBS, Invitrogen) and 1% antibiotic solution (Invitrogen). Each cell line was maintained at 37 °C in a 5% CO_2_ incubator.

### Cell viability and viable cell counts

Cell viability was determined using a Cell Counting Kit-8 Assay Kit (Sigma). HepG2 and Hep3B cells were seeded into 96-well plates at a density of 1 × 10^4^ cells per well and were treated with the indicated concentrations (0–40 *μ*g/ml) of simvastatin for 24 or 48 h. After the cells had incubated, we assessed their viability using the above assay kit, in accordance with the manufacturer's instructions. The absorbance was detected at 450 nm with an ELISA Plate Reader (PerkinElmer, Waltham, MA, USA). To measure and calculate the fractions of dead and live cells, we determined the counts of death cells by Trypan blue exclusion in a hemocytometer. The results were expressed as a percentage of the control.

### DNA content assay

Cell cycle distributions were determined by DNA content assay. HepG2 and Hep3B cells were seeded into six-well plates and treated with the indicated concentrations (0-20 *μ*g/ml) of simvastatin for 48 h. At incubation time, the cells were harvested and fixed in 70% ethanol at 4 °C overnight, and then they were washed with phosphate-buffered saline (PBS) and stained with 20 *μ*g/ml PI at 37 °C for 30 min. The cell populations were analyzed by FAC Sort flow cytometry (BD Biosciences, San Jose, CA, USA).

### Reverse transcription polymerase chain reaction (RT-PCR) and real-time PCR

In all, 1*μ*g of total RNA from the culture cells was extracted using Trizol Reagent (Invitrogen). Complementary DNA (cDNA) synthesized with the transcriptor first strand cDNA synthesis kit (Clontech, Mountain View, CA, USA) according to the manufacturer's instructions. To amplify the target genes, PCR was performed using specific primers: p21, forward 5′-GAGCGATGGAACTTCGACTT-3′ and reverse 5′-GGCGTTTGGAGTGGTAGAAA-3′ p27, forward 5′-TCTACTGCGTGGCTTGTCAG-3′ and reverse 5′-CTGTATTTGGAGGCACAGCA-3′ Skp2, forward 5′-TGAGCTGCTCTTGGGAATCT-3′ and reverse 5′-GTCTGGGACAGCTGCTTAGG-3′ GAPDH as endogenous control, forward 5′-ACCACAGTCCATGCCATCAC-3′ and reverse 5′-TCCACCACCCTGTTGCTGTA-3′. PCR amplification used TEMPase Hot Start DNA Polymerase (Ampliqon, Hamburg, Germany). Procedures were described as follows: 50 ng of cDNA was added to 20 *μ*l of PCR mixture. The PCR mixture was performed after 15 min of denaturation at 95  °C and then amplification for 25 cycles contained the 15 s of denaturation at 95 °C, 30 s of annealing step at 60 °C, 60 s of extension at 72 °C, and another 10 min at 72 °C after the last cycle. The PCR products were separated on 2% agarose gels. For real-time PCR analysis, the synthesized cDNAs were mixed with 2X SYBR Green PCR Master Mix (Applied Biosystems, Foster City, CA, USA) were detected by the StepOnePlus Real-Time PCR System (Applied Biosystems). A pair of gene-specific forward and reverse primers (p21: 5′-AGACTCTCAGGGTCGAAAAC-3′, 5′-TGGAGTGGTAGAAATCTGTCATG-3′ p27: 5′-TGCAACCGACGATTCTTCTAC-3′, 5′-CTTCTGTTCTGTTGGCTCTTTTG-3′ Skp2: 5′-CTGTCTCAAGGGGTGATTGC-3′, 5′-TTCGATAGGTCCATGTGCTG-3′ GAPDH: 5′-ACCACAGTCCATGCATCAC-3′ 5′-TCCACCACCCTGTTGCTGT-3′). All reactions were performed in triplicate. The relative amounts of mRNAs were calculated using the comparative CT method. Human GAPDH mRNA was used as the internal control.

### siRNA transfections

Human AMPK (siRNAs; Santa Cruz), p21, p27 and control siRNAs (GE Dharmacon, Lafayette, CO, USA) were transiently transfected into cells with INTERFERin transfection reagent according to the manufacturer's instructions (Polyplus Transfection, New York, NY, USA). After 24 h, the cells were treated with simvastatin (0 or 10 *μ*g/ml) for 48 h and harvested for immunoblotting or DNA content assay.

### Plasmid DNA transfections

293T cells were plated at a density of 1 × 10^5^ cells per well in six-well plates overnight. The cells were transfected using JetPEI transfection reagent (Polyplus Transfection), according to the retrovirus manufacturer's instructions, with a lenti- or retroviral vector encoding constitutively activated STAT3-GFP (EF.STAT3C.Ubc.GFP, Addgene plasmid 24983, Addgene, Cambridge, MA, USA)^57^ or p-Babe-N-tag-SKP2^58^ and expression vectors encoding the packing proteins *gag-pol* and *VSV-G*. Viral supernatants were collected starting 48 and 96 h after transfection and filtered through a 0.45-*μ*m filter. Then, for viral transduction processing, we added HepG2 cells to infecting medium with 8 *μ*g/ml polybrene for 48 h. The HepG2 cells were subsequently treated with simvastatin (0 or 20 *μ*g/ml) for 48 h and harvested for immunoblotting or cell cycle analysis.

### Luciferase assay

Cells were co-transfected with a pGL4.18-SKP2 promoter reporter plasmid^58^ and a control Renilla luciferase reporter plasmid (Promega, Madison, WI, USA) for 24 h. At the indicated time, the cells were treated with simvastatin 20 *μ*g/ml for 24 h and then harvested. Luciferase activity was determined using a dual-luciferase reporter assay system (Promega). Light units were normalized to Renilla luciferase activity.

### Immunoblotting

After the indicated treatments, cells were harvested and lysed in PRO-PREP protein extraction solution (iNtRON, Taipei, Taiwan) containing a protease inhibitor cocktail. Cell lysates were centrifuged at 12 000* g* for 15 min at 4 °C, and the supernatants were collected. The protein concentration of the samples was measured using a Bio-Rad protein assay kit (Bio-Rad, Hercules, CA, USA). Then, 40–50 *μ*g protein from each sample was separated on a 10% or 12% SDS-polyacrylamide gel before being transferred onto equilibrated polyvinylidene difluoride membranes. After being blocked, the membranes were incubated with the appropriate primary antibodies at 4 °C overnight. Then, membranes were incubated with the appropriate secondary antibodies at 4 °C for 2 h, and the signals were detected using an Odyssey Imaging System (Odyssey, Lincoln, NE, USA). *β*-Actin was used as a loading control in the immunoblotting analysis.

### Tumor growth in the xenograft mouse model

Male BALB/c nude mice (6–8 weeks old) were purchased from the National Laboratory Animal Center (NLAC, Taipei, Taiwan). HepG2 cells were cultured with DMEM supplemented with 10% FBS. Viable HepG2 cells (1.0 × 10^7^ cells in 0.2 ml of serum-free DMEM) were injected subcutaneously into the upper right flanks of the mice. Treatment was initiated when tumor volumes reached a mean size of approximately 100 mm^3^. The mice were subsequently subjected to intraperitoneal injections of saline or simvastatin (20 mg/kg body weight) twice a day for 2 weeks. Tumor volumes were measured every other day with calipers and calculated using the following formula: largest diameter × (smallest diameter)^2^ × 0.5. After being treated for 14 days, the mice were killed. The tumors were collected, and the weight of each tumor was measured. All animal care and experimental procedures were approved and conducted by the Committee for Animal Experiments, National Chung Hsing University, Taichung, Taiwan (approved document La-1051401).

### Histological analysis and IHC

Tumor tissues from the HepG2 tumor-bearing control mice and simvastatin treatment mice were harvested, fixed in 10% formalin and embedded in paraffin. The paraffin sections were subsequently stained with hematoxylin and eosin for morphological observation. For IHC, the paraffin slides were deparaffinized and rehydrated in xylene and ethanol. Antigen retrieval was performed in Tris/EDTA (pH 9.0) buffer by heating for 30 min, followed by incubation in an enzymatic antigen retrieval solution for 10 mm for 20 min. The slides were incubated with 0.3% H_2_O_2_ in TBS for 15 min and washed with 0.025% Triton X-100 in TBS and then blocked with 1% BSA in TBS for 2 h at room temperature before being incubated at 4 °C overnight with the appropriate primary antibody. Then, the slides were incubated with the appropriate HRP-conjugated secondary antibody for 1 h at room temperature and visualized using 3,3' diaminobenzidine substrate and counterstaining with hematoxylin. Images of the slides were acquired using inverted microscopy.

### Statistical analysis

All assays were performed as three independent experiments in duplicate or triplicate. Data were analyzed using Student's *t*-test, and significant differences were inferred at a *P*-value of 0.05.
