# Supplementary material for: Simvastatin-induced cell cycle arrest through inhibition of STAT3/SKP2 axis and activation of AMPK to promote p27 and p21 accumulation in hepatocellular carcinoma cells
Source: Cell Death Dis. 2017 Feb 23;8(2):e2626–. doi: 10.1038/cddis.2016.472 (PMC5386458; doi:10.1038/cddis.2016.472)
Supplement: Supplementary Information [file cddis2016472x1.doc]

**Supplementary Data:**

**Materials and methods**

**DNA content assay**

Cell cycle distributions were determined by DNA content assay. HepG2 and Hep3B cells were seeded into 6-well plates and treated with concentrations (0, 40 μg/ml) of simvastatin for 48hours. At incubation time, cells were harvested and fixed in 70% ethanol at 4°C overnight. Cells were washed with phosphate-buffered saline (PBS) and stained with 20 μg/ml PI at 37°C for 30 minutes. The cell populations were analysed by FAC Sort ﬂow cytometry (BD Biosciences, San Jose, California, U.S.A.).

**Western blot**

Control and Skp2-overexpressing HepG2 cells were treated with simvastatin (0, 20 μg/ml) for 12 hours, and then treated with 10 μg/ml cycloheximide (CHX) for 1, 2, 4, 6 or 12 hours. After that, the cell lysates were collected for protein expression detection by immunoblotting using p27 and β-actin antibodies.

**Design of population study**

Study design and study population: Based on Taiwan’s National Health Insurance Research Database (NHIRD), we conducted a retrospective cohort study. We identified all newly diagnosed HCC patients who were registered in the Registry for Catastrophic Illness Patient Database (RCIPD) between January 1, 1997 and December 31, 2012 (ICD-9 codes: 155, 155.0, 155.2). HCC registration in the RCIPD requires histologically approval or typical computerized tomography images for those not suitable for surgery or biopsy. The details of NHIRD and RCIPD have been described in our previous studies ^5,8^. This study has been approved by Institute Review Board of Taiwan’s National Health Research Institutes. To improve comparability, only those who received liver resection as their initial therapy were selected. Patient who did not survive more than 90 days after HCC resection or those with previous malignancies were excluded. The 90 days after liver resection were defined as “landmark period” to classify statins using status. Patients who used statins more than 80 days during the landmark period were defined as statins users. Those used statins less than 2 days were defined as non-users. Each statins user was matched with 4 matched non-users by propensity scores. Propensity scores were defined in prior and calculated based on age, gender, liver resection date, and comorbidities. To avoid immortal time bias, both statins users and non-users were followed up from the first date after landmark period after liver resection until death, up to 5 years, or the end of 2012. Death was defined as withdrawal of the patient from the National Health Insurance program. Cumulative incidence of overall mortality was calculated. The number of patients who needed to be treated for one less death was defined as NNT and calculated by the inverse of the absolute risk reduction. To determine whether statins use was independently associated with reduced risk of overall mortality, Cox proportional hazards model was developed. The demographic characteristics of the statins users and non-users were compared using the χ^2^ test or Student’s t-test. All data management was performed using SAS 9.1 software (SAS Institute, Cary, NC). Cumulative incidences were analyzed using the survival and epitools packages of R.

**Results**

**High dosage simvastatin treatment induces cell apoptosis in HCC cell lines**

To determine the mechanism of simvastatin-treated hepatoma cell apoptosis, we analyzed the effect of simvastatin (0, 40 μg/ml) on cell cycle distribution using flow cytometry. After 48 hours incubation, the sub-G1 cell populations of HepG2 cell was significantly increased in simvastatin-treated cells. The less apoptosis was also found in simvastatin-treated Hep3B cells (**supplemental Figure S1**)

**Skp2 overexpression promoted p27 depletion in simvastatin-treated HepG2 cells.**

To examine whether p27 stability could be mediated by Skp2 during simvastatin stimulation, control and Skp2-overexpressing HepG2 cells were treated with simvastatin (0, 20 μg/ml) for 12 hours, and following treatment with the translation inhibitor cycloheximide (CHX). After that, the cell lysates were collected for protein expression detection by immunoblotting. Compared with control HepG2 cells, Skp2-overexpressing HepG2 cells did not maintain the simvastatin-enhanced p27 protein stability after treatment with CHX (**supplementary Figure S2**).

**Statins use is associated with reduced risk of HCC overall mortality in population study**

Between 1997 and 2012, a total of 79,718 patients were newly diagnosed with HCC. Among them, 13,233 patients had received liver resection as their initial HCC therapy. After excluding those with follow-up less than 90 days or with other malignancies, 10,741 patients were identified. Among the 90 days landmark period after liver resection, 10,348 patients used statins less than 2 days were defined as non-users, and 168 patients used statins more than 80 days were defined as statins users. After propensity score matching with a 1:4 ratio, we finally identified 152 statins users and 608 non-statins users **(supplemental Fig. S4)**. The demographic data before and after propensity score matching was shown in **supplemental Table S1 and S2.**

Cumulative incidences of overall mortality are shown in **Figure S3.** Statins users were associated with significantly lower overall mortality (5-year cumulative incidence: 23.71%; 95% CI, 9.19-38.23%) than non-users (32.57%; 95% CI, 28.09-37.04%) (P=0.005). The unadjusted NNT associated with one less death within 5 years was 12. This suggests that use of statins in 12 HCC patients is associated with one less death within 5 years after liver resection. After adjusting for other confounders, we found that statins use is associated with a significantly lower risk of overall mortality (adjusted HR=0.47; 95% CI, 0.27-0.80, P=0.005). Liver decompensation and vascular invasion were found to be statistically significant risk factors for increased overall mortality **(Supplemental Table S3)**. Therefore, statins use significantly decreased overall mortality and increased survival rate in HCC patients.
