# Supplementary material for: Simvastatin-induced cell cycle arrest through inhibition of STAT3/SKP2 axis and activation of AMPK to promote p27 and p21 accumulation in hepatocellular carcinoma cells
Source: Cell Death Dis. 2017 Feb 23;8(2):e2626–. doi: 10.1038/cddis.2016.472 (PMC5386458; doi:10.1038/cddis.2016.472)
Supplement: Supplementary Figure [file cddis2016472x2.doc]

**Supplementary Figure S1. Simvastatin induces cell apoptosis in HCC cell lines.** The sub-G1 cell populations increased in HCC cells. HepG2 and Hep3B cells were treated with simvastatin (0, 40 μg/ml) for 48 hours, and then cell cycle distributions were analyzed by propidium iodide (PI) staining and flow cytometry.

**Supplementary Figure S2. Skp2 overexpression promoted p27 depletion in simvastatin-treated HepG2 cells.** Control and Skp2-overexpressing HepG2 cells were treated with simvastatin (0 or 20 μg/ml) for 12 hours, and then treated with 10 μg/ml CHX for 1, 2, 4, 6 or 12 hours. After that, the cell lysates were collected for protein expression detection by immunoblotting using p27 and β-actin antibodies.

**Supplementary Figure S3. Cumulative incidences of overall mortality.** Kaplan-Meier method was used to calculate and compare cumulative incidences. Both the statins use cohort and non-users cohort were followed up from the index date, the first day after the 90 days landmark period.

**Supplementary Figure S4. Flowchart for study patient selection.** The sum of case numbers excluded by each individual criterion may outnumber the total excluded cases in each step as a case may be excluded due to more than one criterion.
