# Supplementary figures and images for: Simvastatin-induced cell cycle arrest through inhibition of STAT3/SKP2 axis and activation of AMPK to promote p27 and p21 accumulation in hepatocellular carcinoma cells
Source: Cell Death Dis. 2017 Feb 23;8(2):e2626–. doi: 10.1038/cddis.2016.472 (PMC5386458; doi:10.1038/cddis.2016.472)

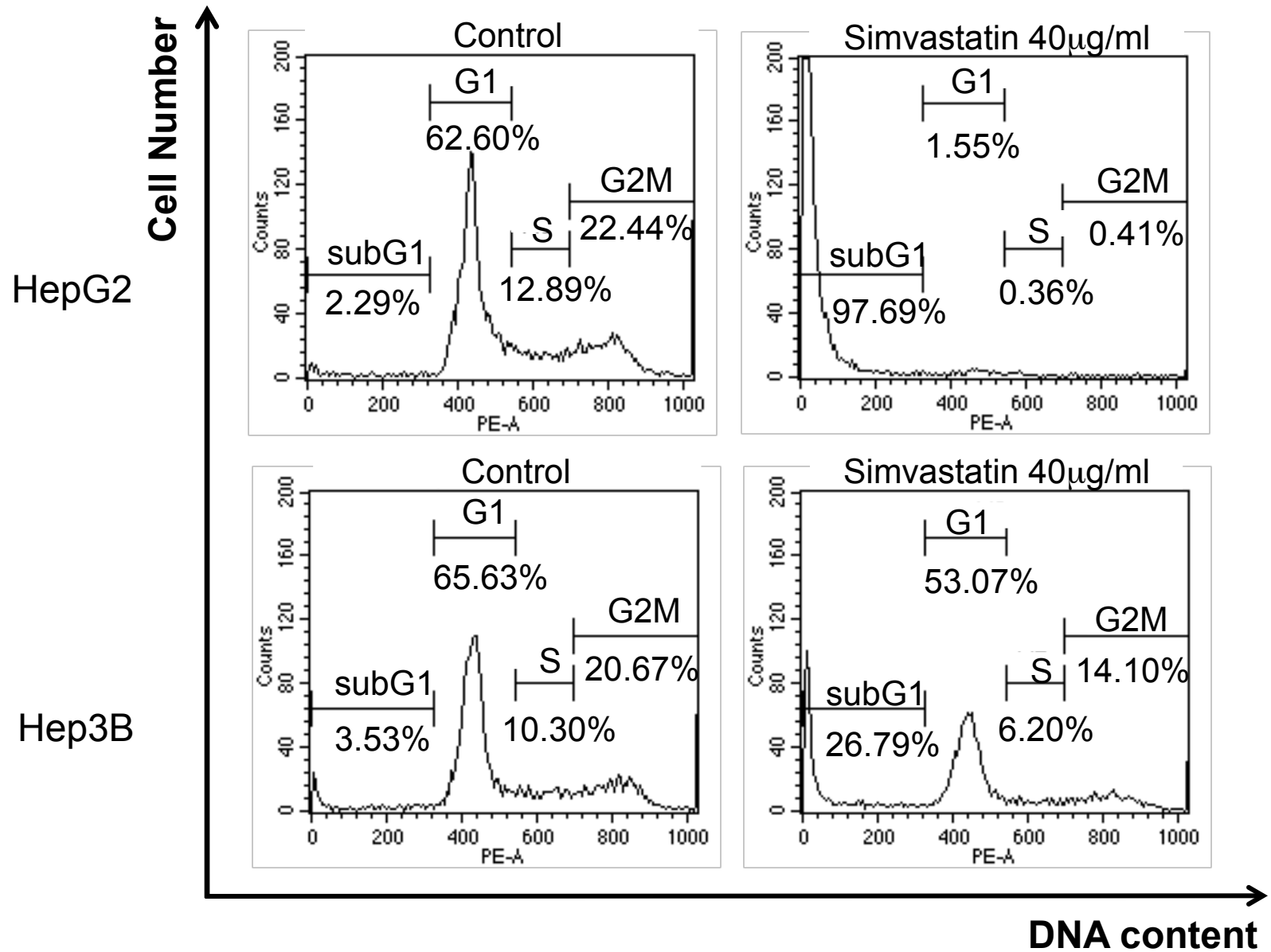

Supplementary Figure S1

Supplement: Supplementary Figure 1 [file cddis2016472x3.pdf]

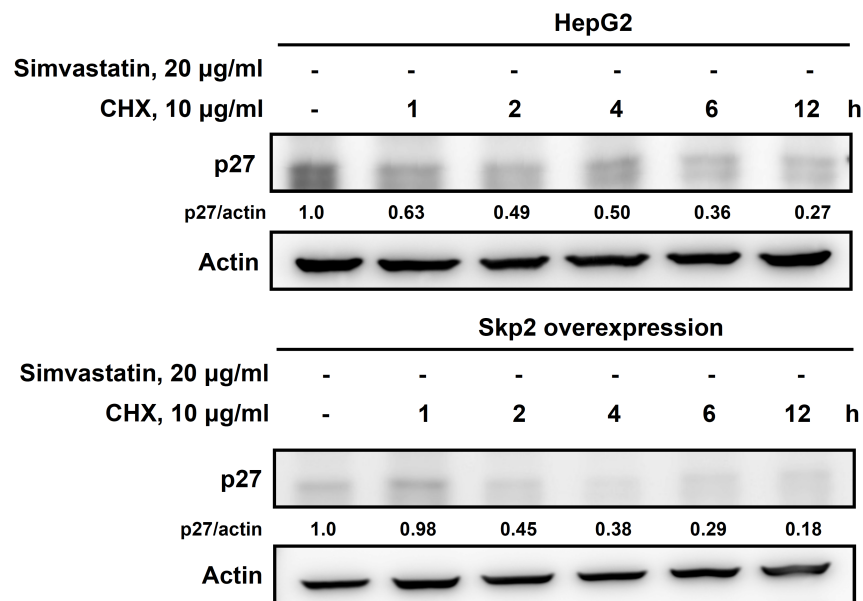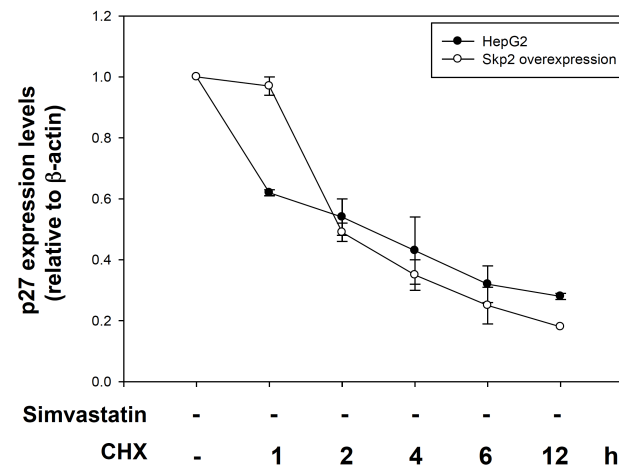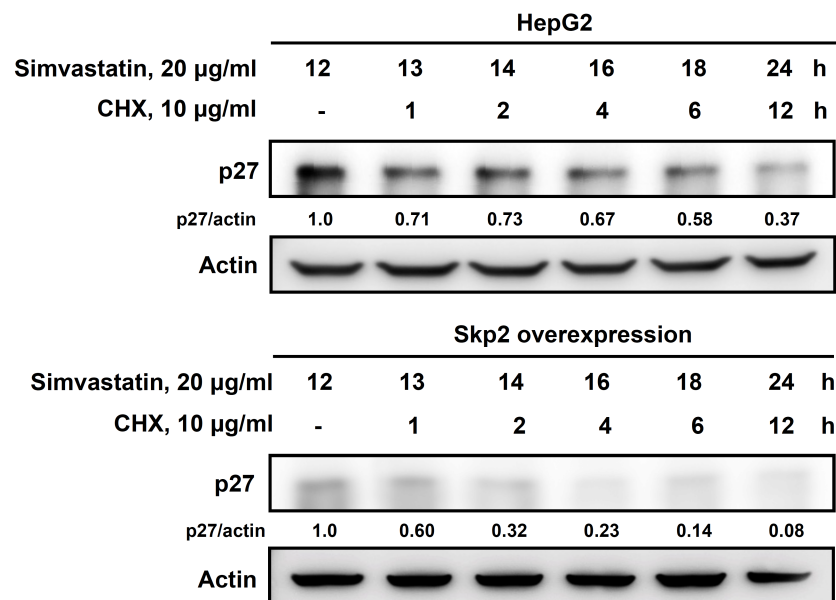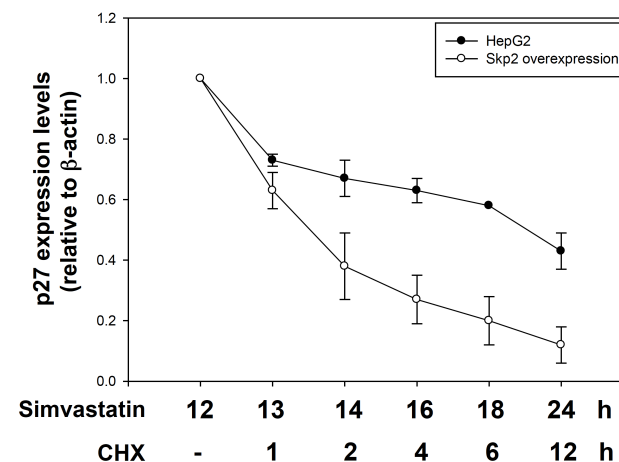

Supplementary Figure S2

Supplement: Supplementary Figure 2 [file cddis2016472x4.pdf]

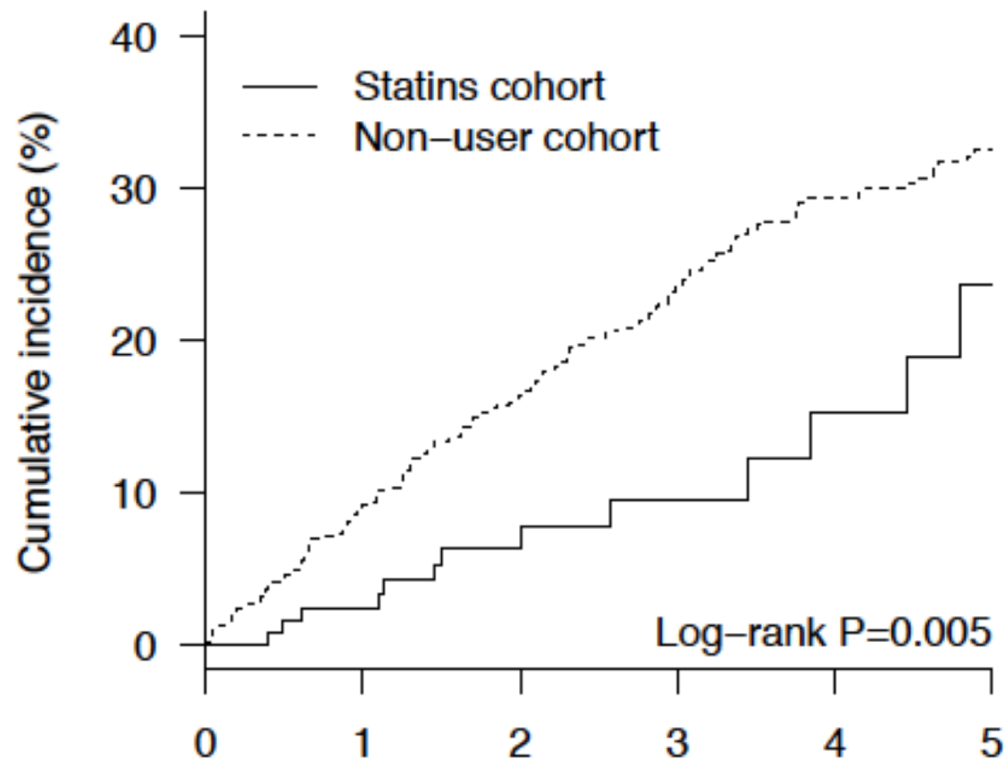

| Number at risk |     |     |     |     |     |     |
|----------------|-----|-----|-----|-----|-----|-----|
|                | 0   | 1   | 2   | 3   | 4   | 5   |
| Statins        | 152 | 106 | 67  | 42  | 27  | 14  |
| Non-user       | 608 | 493 | 407 | 310 | 228 | 173 |

Supplement: Supplementary Figure 3 [file cddis2016472x5.pdf]

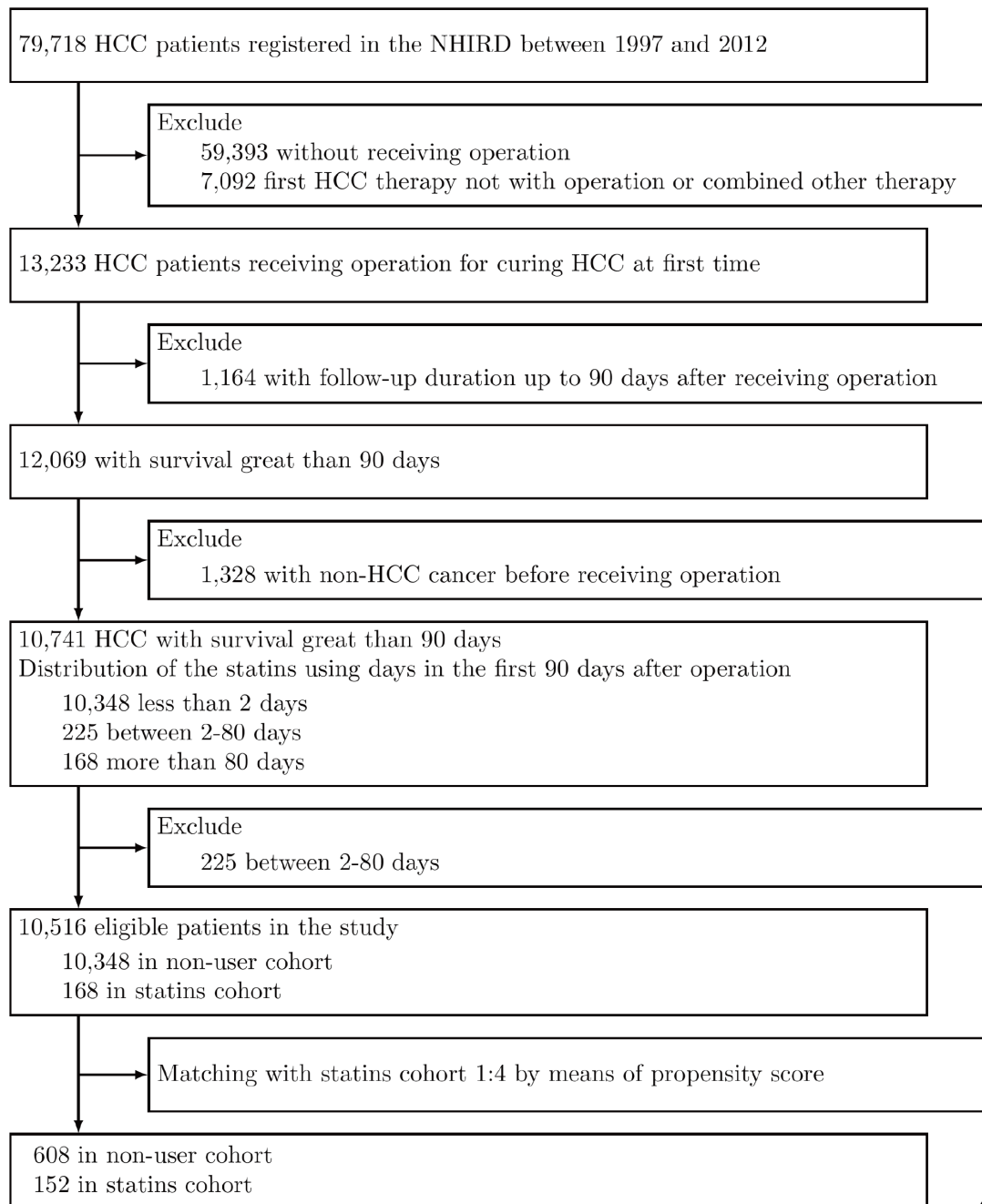

Supplement: Supplementary Figure 4 [file cddis2016472x6.pdf]
