# Supplementary material for: Simvastatin-induced cell cycle arrest through inhibition of STAT3/SKP2 axis and activation of AMPK to promote p27 and p21 accumulation in hepatocellular carcinoma cells
Source: Cell Death Dis. 2017 Feb 23;8(2):e2626–. doi: 10.1038/cddis.2016.472 (PMC5386458; doi:10.1038/cddis.2016.472)
Supplement: Supplementary Table 1 [file cddis2016472x7.doc]

**Supplemental Tables:**

**Supplemental Table S1: Baseline demographic characteristics and outcomes of study cohorts before propensity score matching**

|  | Statins users*  (N=168)  Number (%) | Non-users*  (N=10348)  Number (%) | P value^#^ |
| --- | --- | --- | --- |
| **Age** (mean±SD)^+^ | 65.7±9.6 | 58.7±13.2 | <.001 |
| **Gender** |  |  |  |
| Female | 43 (25.6) | 2608 (25.2) | 0.979 |
| Male | 125 (74.4) | 7740 (74.8) |  |
| **Follow-up years** (mean±SD)^ |  |  |  |
| Mean±SD | 2.3±2.1 | 4.2±3.5 | <.001 |
| Median (IQR) | 1.7 (0.8-3.0) | 3.2 (1.4-6.1) | <.001 |
| **Statins Using days during landmark period** |  |  |  |
| Mean±SD | 88.1±2.8 | 0.0±0.0 | <.001 |
| Median (IQR) | 90.0 (85.0-90.0) | 0.0 (0.0-0.0) | <.001 |
| **Concomitant drug users^++^** |  |  |  |
| Antiviral drug | 20 (11.9) | 2080 (20.1) | 0.011 |
| **Major comorbidities** |  |  |  |
| Hepatitis B virus infection | 70 (41.7) | 5618 (54.3) | 0.001 |
| Hepatitis C virus infection | 50 (29.8) | 3439 (33.2) | 0.387 |
| Liver cirrhosis | 43 (25.6) | 4133 (39.9) | <.001 |
| Acute coronary syndrome | 70 (41.7) | 840 (8.1) | <.001 |
| Cerebral vascular disease | 26 (15.5) | 613 (5.9) | <.001 |
| Chronic obstructive pulmonary disease | 20 (11.9) | 741 (7.2) | 0.028 |
| Diabetes | 114 (67.9) | 2103 (20.3) | <.001 |
| Liver failure | 1 (0.6) | 191 (1.8) | 0.363 |
| Renal failure | 14 (8.3) | 368 (3.6) | 0.002 |
| Hypertension | 132 (78.6) | 3231 (31.2) | <.001 |
| Hyperlipidemia | 16 (9.5) | 125 (1.2) | <.001 |
| Peptic ulcer diseases | 35 (20.8) | 2031 (19.6) | 0.770 |
| Liver decompensation | 4 (2.4) | 491 (4.7) | 0.211 |
| Vascular invasion | 2 (1.2) | 119 (1.1) | >.999 |
| **Propensity Score^##^** |  |  |  |
| Mean±SD | 0.1±0.1 | 0.0±0.0 | <.001 |
| Median (IQR) | 0.1 (0.0-0.1) | 0.0 (0.0-0.0) | <.001 |
| **Events** |  |  |  |
| Death | 20 (11.9) | 2945 (28.5) | <.001 |

*Statins users: receiving statins more than 80 days during the landmark period (the first 90 days after liver resection); Non-users: receiving statins less than 2 days during the landmark period

^#^: P values were compared using the χ^2^ test and Student’s t-test.

^+^: Age is treated as a continuous variable

^: Follow-up starts since the first day after the landmark period.

^++^: Drug users indicate patients using drugs at least one day per month on average.

**^##^**: Age, gender, acute coronary syndrome, cerebral vascular diseases, COPD, diabetes, cirrhosis, liver decompensation, renal failure, hypertension, hypercholesterolemia, use of antiviral therapy were included in the propensity score calculation.

Abbreviations: N, number; SD: standard deviation; IQR: interquartile range
