# Supplementary material for: Simvastatin-induced cell cycle arrest through inhibition of STAT3/SKP2 axis and activation of AMPK to promote p27 and p21 accumulation in hepatocellular carcinoma cells
Source: Cell Death Dis. 2017 Feb 23;8(2):e2626–. doi: 10.1038/cddis.2016.472 (PMC5386458; doi:10.1038/cddis.2016.472)
Supplement: Supplementary Table 2 [file cddis2016472x8.doc]

**Supplemental Table S2: Baseline demographic characteristics and outcomes of study cohorts after propensity score matching**

|  | Statins users*  (N=152)  Number (%) | Non-users*  (N=608)  Number (%) | P value^#^ |
| --- | --- | --- | --- |
| **Age** (mean±SD)^+^ | 65.5±9.7 | 65.1±9.3 | 0.687 |
| **Gender** |  |  |  |
| Female | 40 (26.3) | 166 (27.3) | 0.886 |
| Male | 112 (73.7) | 442 (72.7) |  |
| **Follow-up years** (mean±SD)^ |  |  |  |
| Mean±SD | 2.4±2.2 | 3.7±2.9 | <.001 |
| Median (IQR) | 1.8 (0.8-3.1) | 3.1 (1.4-5.3) | <.001 |
| **Statins Using days during landmark period** |  |  |  |
| Mean±SD | 88.1±2.8 | 0.0±0.0 | <.001 |
| Median (IQR) | 90.0 (84.0-90.0) | 0.0 (0.0-0.0) | <.001 |
| **Concomitant drug users^++^** |  |  |  |
| Antiviral drug | 19 (12.5) | 76 (12.5) | >.999 |
| **Major coexisting diseases** |  |  |  |
| Hepatitis B virus infection | 65 (42.8) | 248 (40.8) | 0.726 |
| Hepatitis C virus infection | 48 (31.6) | 194 (31.9) | >.999 |
| Liver cirrhosis | 42 (27.6) | 170 (28.0) | >.999 |
| Acute coronary syndrome | 55 (36.2) | 213 (35.0) | 0.864 |
| Cerebral vascular disease | 22 (14.5) | 88 (14.5) | >.999 |
| Chronic obstructive pulmonary disease | 18 (11.8) | 57 (9.4) | 0.447 |
| Diabetes | 98 (64.5) | 396 (65.1) | 0.955 |
| Liver failure | 1 (0.7) | 7 (1.2) | 0.929 |
| Renal failure | 12 (7.9) | 46 (7.6) | >.999 |
| Hypertension | 116 (76.3) | 474 (78.0) | 0.744 |
| Hyperlipidemia | 8 (5.3) | 25 (4.1) | 0.689 |
| Peptic ulcer diseases | 31 (20.4) | 126 (20.7) | >.999 |
| Liver decompensation | 4 (2.6) | 17 (2.8) | >.999 |
| Vascular invasion | 1 (0.7) | 4 (0.7) | >.999 |
| **Propensity Score^##^** |  |  |  |
| Mean±SD | 0.1±0.1 | 0.1±0.1 | 0.982 |
| Median (IQR) | 0.0 (0.0-0.1) | 0.0 (0.0-0.1) | 0.992 |
| **Events** |  |  |  |
| Death | 15 (9.9) | 191 (31.4) | <.001 |

*Statins users: receiving statins more than 80 days during the landmark period (the first 90 days after liver resection); Non-users: receiving statins less than 2 days during the landmark period

^#^: P values were compared using the χ^2^ test and Student’s t-test.

^+^: Age is treated as a continuous variable

^: Follow-up starts since the first day after the landmark period.

^++^: Drug users indicate patients using drugs at least one day per month on average.

**^##^**: Age, gender, acute coronary syndrome, cerebral vascular diseases, COPD, diabetes, cirrhosis, liver decompensation, renal failure, hypertension, hypercholesterolemia, use of antiviral therapy were included in the propensity score calculation.

Abbreviations: N, number; SD: standard deviation; IQR: interquartile range
