# Supplementary material for: Simvastatin-induced cell cycle arrest through inhibition of STAT3/SKP2 axis and activation of AMPK to promote p27 and p21 accumulation in hepatocellular carcinoma cells
Source: Cell Death Dis. 2017 Feb 23;8(2):e2626–. doi: 10.1038/cddis.2016.472 (PMC5386458; doi:10.1038/cddis.2016.472)
Supplement: Supplementary Table 3 [file cddis2016472x9.doc]

**Supplemental Table S3:**

**Multivariable Cox proportional hazards model analysis for risk of overall mortality**

|  | Patients (Number) | Events (Number) | HR | 95% CI | p-value |
| --- | --- | --- | --- | --- | --- |
| Statins users vs. non-users | 152 | 15 | 0.47 | 0.27-0.80 | 0.005 |
| Age, per each incremental year | 760 | 206 | 1.01 | 1.00-1.03 | 0.114 |
| Male | 554 | 155 | 1.22 | 0.88-1.71 | 0.230 |
| Hepatitis B virus infection | 313 | 83 | 1.12 | 0.79-1.58 | 0.536 |
| Hepatitis C virus infection | 242 | 70 | 1.05 | 0.76-1.46 | 0.748 |
| Liver cirrhosis | 212 | 66 | 1.05 | 0.75-1.48 | 0.766 |
| Acute coronary syndrome | 268 | 62 | 0.85 | 0.61-1.17 | 0.306 |
| Cerebral vascular disease | 110 | 28 | 1.22 | 0.80-1.88 | 0.356 |
| Chronic obstructive pulmonary disease | 75 | 17 | 1.10 | 0.65-1.86 | 0.728 |
| Diabetes | 494 | 132 | 1.21 | 0.90-1.64 | 0.214 |
| Liver failure | 8 | 2 | 0.83 | 0.19-3.72 | 0.808 |
| Renal failure | 58 | 17 | 1.64 | 0.95-2.82 | 0.075 |
| Hypertension | 590 | 164 | 1.44 | 0.98-2.11 | 0.066 |
| Hypercholesterolemia | 33 | 6 | 0.55 | 0.23-1.30 | 0.174 |
| Peptic ulcer | 157 | 47 | 1.23 | 0.87-1.75 | 0.247 |
| Liver decompensation | 21 | 11 | 2.37 | 1.16-4.84 | 0.018 |
| Vascular invasion | 5 | 3 | 4.16 | 1.25-13.85 | 0.020 |
| Antiviral drug | 95 | 16 | 0.64 | 0.37-1.09 | 0.099 |
